# Supplementary figures and images for: Individual response to mTOR inhibition in delaying replicative senescence of mesenchymal stromal cells
Source: PLoS One. 2019 Jan 31;14(1):e0204784. doi: 10.1371/journal.pone.0204784 (PMC6354956; doi:10.1371/journal.pone.0204784)

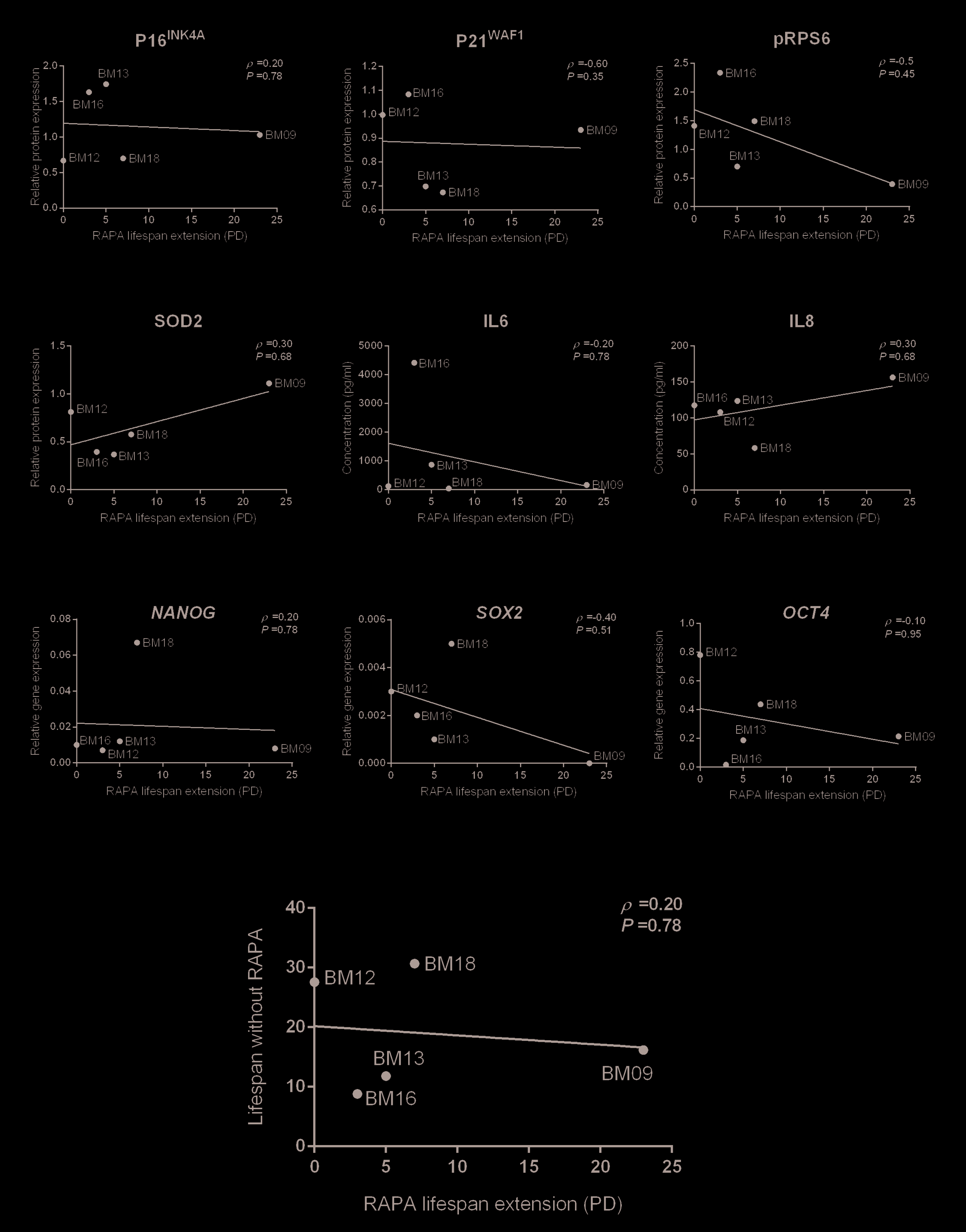

Supplement: S1 Fig — The normalized expression values of senescence-associated proteins (p16INK4A, p21WAF1, pRPS6, SOD2, IL6 and IL8) and pluripotency-related genes (NANOG, SOX2 and OCT4) in each BM-MSC sample at early passages (5th and 6th passages) (previously determined in Piccinato et al., 2015) [14] (A), as well the final PD number of each BM-MSC sample expanded in normal medium (without rapamycin) (B), were plotted against the additional PD number obtained for the corresponding rapamycin-treated cells and statistically analyzed by Spearman correlation, as shown in the graphs. RAPA = rapamycin. PD = population doubling. (TIF) [file pone.0204784.s001.tif]
